# Vacuum-Based Impregnation of Liquid Glass into Sunflower Press Cake Particles and Their Use in Bio-Based Rigid Polyurethane Foam

**DOI:** 10.3390/ma14185351

**Published:** 2021-09-16

**Authors:** Agnė Kairytė, Sylwia Członka, Renata Boris, Sigitas Vėjelis

**Affiliations:** 1 Laboratory of Thermal Insulating Materials and Acoustics, Institute of Building Materials, Faculty of Civil Engineering, Vilnius Gediminas Technical University, Linkmenu st. 28, LT-08217 Vilnius, Lithuania; sigitas.vejelis@vilniustech.lt; 2 Institute of Polymer & Dye Technology, Lodz University of Technology, 90-924 Lodz, Poland; sylwia.czlonka@dokt.p.lodz.pl; 3 Laboratory of Composite Materials, Institute of Building Materials, Faculty of Civil Engineering, Vilnius Gediminas Technical University, Linkmenu st. 28, LT-08217 Vilnius, Lithuania; renata.boris@vilniustech.lt

**Keywords:** sunflower press cake, bio-polyurethane foam, liquid glass, thermal insulation, circular economy, mechanical performance, water absorption

## Abstract

The study analyses rigid polyurethane (PUR) foam modified with 10–30 wt.% sunflower press cake (SFP) and liquid glass-impregnated sunflower press cake (LG-SFP) particles and their impact on performance characteristics of PUR foams—foaming behaviour, rheology, thermal conductivity, compressive strength parallel and perpendicular to the foaming directions, tensile strength, dimensional stability, short-term water absorption by partial immersion, and thermal stability. Even though the dynamic viscosity and apparent density were increased for SFP and LG-SFP formulations, thermal conductivity values improved by 17% and 10%, respectively, when 30 wt.% of particles were incorporated. The addition of SFP and LG-SFP particles resulted in the formation of more structurally and dimensionally stable PUR foams with a smaller average cell size and a greater content of closed cells. At 30 wt.% of SFP and LG-SFP particles, compressive strength increased by 114% and 46% in the perpendicular direction, respectively, and by 71% and 67% in the parallel direction, respectively, while tensile strength showed an 89% and 85% higher performance at 30 wt.% SFP and LG-SFP particles loading. Furthermore, short-term water absorption for all SFP and LG-SFP modified PUR foam formulations was almost two times lower compared to the control foam. SFP particles reduced the thermal stability of modified PUR foams, but LG-SFP particles shifted the thermal decomposition temperatures towards higher ones.

## 1. Introduction

The building sector significantly affects the economy and environment. This sector is the main consumer of approximately 36% of the total energy and 50% of the total raw materials used [1,2]. Since the building sector accounts for 39% of all energy and technology-related carbon dioxide emissions, further emissions of greenhouse gas will significantly contribute to upcoming environmental issues.

Currently, eco-consciousness has increased the interest in circular economy and sustainable development goals, including industrial ecology, sustainability, and environmental protection. The reduction of dependence on petrochemical resources is one of their goals. Composite materials are the most interesting solution because they offer reduced carbon dioxide emissions, new life for waste-based materials as constituents for the production of composites, increased lifespan of the final products through durability and resistance to environmental impact, and reduced costs of the final products, etc. Recently, the development of filler application techniques in composites blends has become driven by an aspiration to enhance the properties of the materials, reduce the cost of production, and improve the physical and mechanical performance. Composite materials consist of a filler and matrix, which are significantly important in the synthesis of composites. The purpose of the fillers is to reinforce and modify products, thus replacing petroleum-based polymers in composite materials. Natural or plant-based materials include reinforcing particles or fibres [3,4,5]. Even though the incorporation of natural fibres and fillers into polymer systems shows great advantages in the performance of the products, the greater contribution of waste-based natural resources on sustainability and circular economy is proven as well.

Polyurethane foam is the most versatile product in the market of building materials because it offers a wide range of properties, such as low apparent density, superior thermal insulation, sufficient resistance to the environmental impact, and high mechanical performance. Due to its versatility, it becomes an exceptional material for the production of a great variety of products, including thermal insulation, automotive seats, furniture, structural parts, refrigeration equipment, etc. Therefore, polyurethane foam becomes one of the most suitable polymeric materials for modification with waste-based natural fillers.

Husainie et al. [6] reported that 1%, 2.5%, and 5% of hazelnut shell and eggshell fillers stimulated the formation of a more uniform structure of rigid polyurethane (PUR) foams, significantly improved tensile strength at 1% filler loading, and produced good elongation properties. Additionally, Członka et al. [7] showed that the addition of 0.1% of keratin feather was beneficial to thermal insulating and mechanical properties of PUR foams, i.e., a 9% decrease in thermal conductivity and a 20% increase in compressive strength were observed. Moreover, the study by Tiuc et al. [8] analysed the impact of fir and birch sawdust on the sound absorption ability of PUR foam composites. They concluded that better sound absorption properties were observed for particles with the size of 4 mm. The literature review proved the positive impact of waste-based natural fillers on PUR foams and their performance characteristics.

Sunflower press cake particles can be considered as another promising filler. Some studies have shown that they could be successfully utilised as animal nutrition due to high amounts of protein, hemicellulose, and cellulose, as a fertilizer that contains minerals such as Mg, Ca, Fe, P, and Mn, or for energy purposes [9]. However, the literature also indicates that the application of oil-based press cakes as food for humans or animals is limited due to anti-nutritional factors [10]. As sunflower oil is a semi-drying oil and sunflower press cake contains up to 15% of oil and >50% insoluble components, such as lignin, cellulose, and hemicellulose [11], it may markedly contribute to a significant improvement in mechanical and water resistance properties of PUR foams.

Therefore, the goal of the current study was to analyse the impact of sunflower press cake (SFP) particles, and liquid glass (LG) impregnated SFP particles on the performance characteristics of rapeseed oil-based PUR foam. Moreover, rheological and structural characterisation, foaming process evaluation, determination of thermal insulation, dimensional stability, water resistance, thermal stability, and mechanical performance were conducted.

## 2. Materials and Methods

### 2.1. Raw Materials

As the first main components, two polyols were used as follows: polyol BioPolyol RD was synthesised from rapeseed oil and purchased from SIA PolyLabs, Riga, Latvia, while polyol petol (hydroxyl value 350 mg KOH/g, water content < 0.2%) and polyol PZ 400-4G (hydroxyl value 421 mg KOH/g, water content < 0.1%) was used to obtain dimensionally and structurally stable foams; it was purchased from Oltchim, Râmnicu Vâlcea, Romania. The second main component was polymeric 4,4′ -diphenylmethane diisocyanate (31.5% NCO) Lupranat M20S (BASF, Ludwigshafen, Germany). Distilled water was implemented as a blowing agent in PUR-REF, SFP, and LG-SFP modified foams formulations. Blowing and gelling reactions were catalysed using a Polycat 9 catalyst from Air Products and Chemicals, Inc., Allentown, PA, USA. A stabilised structure was achieved with silicone surfactant ST-52 which was purchased from Shijiazhuang Chuanghong Technology Co., Ltd., Shijiazhuang, China. SFP filler was supplied by a local company (Vilnius, Lithuania). LG with the density of 0.80 g/cm^3^, purity of 99%, mass fraction of Na_2_O—29%, and mass fraction of SiO_2_—29% was supplied by JSC Lerochemas, Klaipėda, Lithuania.

### 2.2. Preparation of Modified PUR Foams

Before its use, SFP was crushed and milled. The obtained SFP particles were dried at 110 °C temperature for 24 h to remove the excess moisture. Then, the whole mass of SFP particles was divided into two parts. The first part was titled SFP particles, and the second part was vacuum-impregnated with LG and titled LG-SFP particles. Vacuum impregnation was carried out for five cycles with holding at 1 bar for 10 min. Furthermore, the whole particle mass was delivered for thermal treatment at 150 °C for 24 h.

The amounts of materials used for the preparation of PUR-REF, SFP, and LG-SFP particles modified PUR foams are presented in Table 1. PUR-REF, SFP, and LG-SFP particles modified PUR foams were produced as a two-component system with an isocyanate index equal to 125. The system of two polyols was used to achieve dimensionally and structurally stable PUR foams.

The mixture of two polyols was prepared with water, catalyst, and surfactant, then mixed at 1800 rpm speed for 1 min. After the preparation of the premix, 10 wt.%, 20 wt.%, and 30 wt.% SFP and LG-SFP particles were added. Finally, the selected amount of isocyanate was poured into the prepared polyols premix and mixed for another 10 s at 1800 rpm speed. Then, the mass was immediately poured into the open mould and left to cure at (23 ± 2) °C.

### 2.3. Characterisation Methodology

The residual moisture content of SFP and LG-SFP particles was determined based on [12] requirements after drying them at (105 ± 2) °C in a ventilated oven until the weight changes between three weighings were less than 0.1%. Three samples were tested in order to get an average value.

The bulk density of SFP and LG-SFP particles was determined by weighing the 1 L dish which was freely filled with particles from no higher than 5 cm in height. The surplus particles were removed, and the surface was smoothed using a metal ruler. The dish with particles was weighed with an accuracy of 0.01 g, the bulk density was calculated according to the following Equation (1):

(1) $$\rho_{b}=\frac{m_{2}-m_{1}}{V}$$

where $\rho_{b}$ is the bulk density of particles, kg/m^3^; $m_{2}$ is the weight of particles and the dish, kg; $m_{1}$ is the weight of an empty dish, kg; and V is the volume of the dish, m^3^.

The microstructural examination of SFP particles, LG-SFP particles, PUR-REF, SFP, and LG-SFP particles, and modified PUR foams was done with the scanning electron microscope (SEM) JEOL SM–7600F (JEOL Ltd., Tokyo, Japan). Before microstructural analysis, the samples were coated with a gold layer under vacuum using sputter coating machine QUORUM Q150R ES (Quorum Technologies Ltd., East Sussex, United Kingdom). During the analysis of SFP and LG-SFP particles, 4 kV voltage and 11 mm distance to the sample surface were used, while for modified PUR foams the parameters were 4 kV and 10 mm, respectively. For SEM imaging of unmodified and modified PUR foams, samples were prepared from the middle part of the product. In order to evaluate the effectiveness of SFP impregnation with LG, the energy dispersion X-ray spectrometer X-Max was used. The local chemical composition was determined according to characteristic X-ray spectra stimulated by the electron beam. The obtained microstructural SEM images were further used in order to analyse structural parameters such as average cell size and the size of SFP and LG-SFP particles with the software ImageJ. In order to obtain the average value of cell size, three samples were tested.

The measurements of dynamic viscosity were made with shear meter SV-10 (A&D Company Ltd., Tokyo, Japan), which has 0.01 mPa·s accuracy and up to 1200 mPa·s measuring range. The dynamic viscosity was determined at 25 °C temperature for three unmodified SFP and LG-SFP modified premixes.

The characteristic foaming parameters were measured in accordance with [13], Annex E. Before the test, all raw materials were conditioned at (20 ± 1) °C as stated in a product standard. The time was fixed using an electronic stopwatch, which has 0.5 s accuracy.

The apparent density was measured and calculated based on requirements of [14] for ten 100 × 100 mm-sized samples.

The dimensional stability was determined according to [15] at (70 ± 2) °C and (90 ± 5)% relative humidity as well as at (−20 ± 2) °C for samples with the size of 200 × 200 mm after keeping the extreme conditions for 48 h. Before each test, all samples were conditioned to equilibrium with an atmosphere at (23 ± 2) °C and (50 ± 5)% relative air humidity until the changes between two consecutive measurements in length and width directions did not exceed 0.1%. In order to obtain an average value, three samples of each composition for each testing condition were used.

Thermal conductivity was conducted for samples with the size of 300 × 300 mm using the methodology presented in [16] with a heat flow meter FOX 304 with active edge insulation (TA Instruments, Eden Prairie, MN, USA). During the test, the direction of heat flow was upwards. Measurements were done at an average test temperature of 10 °C while the difference between cold and hot plates was 20 °C. Before the test, all samples were conditioned at (23 ± 3) °C and (50 ± 10) °C relative air humidity for 16 h as indicated in the harmonised product standard [13], Annex C. In order to obtain an average value three samples were tested.

The percentage of closed cells was measured and calculated according to [17], method 2 for three samples with the size of 100 × 30 × 30 mm. Testing was carried out at (23 ± 2) °C and (50 ± 5) °C relative air humidity conditions. Before the test, all samples were conditioned for 16 h at (23 ± 2) °C and (50 ± 5)% relative air humidity.

The short-term water absorption was conducted on four 200 × 200 mm-sized samples according to [18], method B. Testing was carried out for 24 h at (23 ± 5) °C water. The samples were placed in water tanks in such a position that they were partially immersed in tap water with their bottom faces (10 ± 2) mm below the upper water level. After 10 s, samples were removed from water, placed into a plastic tray of known weight and weighed. Then, the samples were immersed in water tanks again and left for 24 h. After the test, samples were removed from the tap water and placed into a plastic tray of known weight and weighed again. The water uptake was calculated according to the following Equation (2):

(2) $$m_{24}=\frac{m_{1}-m_{0}}{A}$$

where: $m_{24}$ is short-term water absorption after partial immersion, kg/m^2^; $m_{1}$ is the weight of sample including initial water uptake, kg; $m_{0}$ is the initial water uptake after 10 s, kg; and A is the bottom surface area of the sample, m^2^. Before the test, all samples were conditioned for 6 h at (23 ± 5) °C.

The compressive and tensile strengths were determined based on the requirements of [19] and [20], respectively. The universal testing machine H10KS Hounsfield (Tinius Olsen Ltd., Surrey, United Kingdom) was used to determine the mechanical performance of PUR-REF, SFP, and LG-SFP particles modified PUR foams. Five samples with the size of 50 × 50 mm were used for each test. Both tests were carried out at (23 ± 5) °C and all samples were conditioned for 6 h at (23 ± 5) °C. For the compressive strength test, samples were centrally placed between two plates of the compression machine and preloaded with a pressure of (250 ± 10) Pa. Then, the samples were compressed with the movable plate at a constant displacement rate of 0.1·d per minute (where d is the thickness of the sample). Normalised compressive strength was calculated according to the following Equation (3) [21]:

(3) $$\sigma_{Norm.}=\frac{\rho_{n}\cdot \sigma_{m}}{\rho_{m}}$$

where $\sigma_{Norm.}$ is the normalised compressive strength, kPa; $\rho_{n}$ is the nominal apparent density of PUR foam (in this study, 35 kg/m^3^ apparent density was chosen), kg/m^3^; $\sigma_{m}$ is the measured compressive strength, kPa; and $\rho_{m}$ is the measured apparent density, kg/m^3^.

For tensile strength tests, the samples were attached to two metal plates with two component epoxy glue. The whole assembly was additionally conditioned for more than 6 h at (23 ± 5) °C temperature conditions. Then, the whole assembly was fixed into a universal testing machine and tensile force at a constant speed was applied until failure occurred.

The thermal properties of polyurethane foams were determined by thermogravimetric analysis (TGA) with STA 449 F1 Jupiter Analyser (Netzsch Group, Selb, Germany). The measurement was performed for samples of 10 mg. Samples were heated at 10 °C/min speed in an argon atmosphere in the temperature range of (25–600) °C. The decomposition temperatures—T_5%_ and T_50%_ were determined.

## 3. Results and Discussion

**Figure 1:**
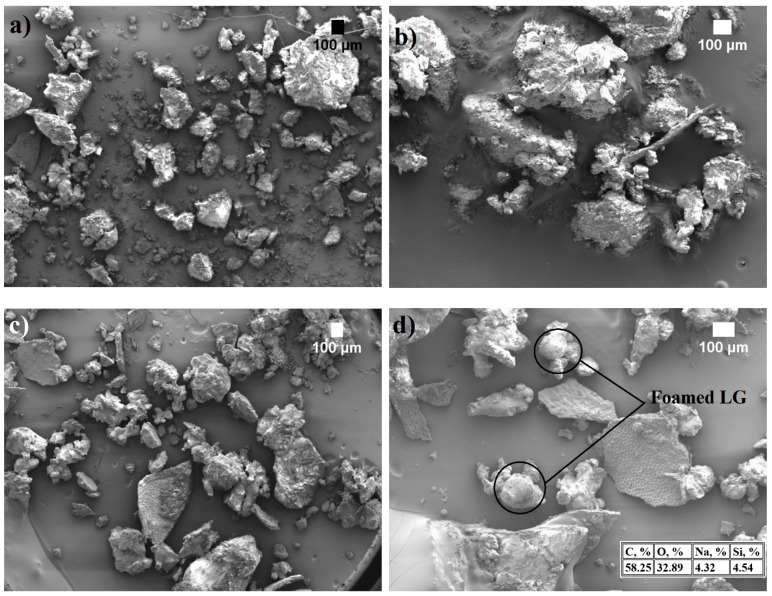
SEM images of particles’ microstructure: (**a**) SFP (x35 magnification); (**b**) SFP (× 60 magnification); (**c**) LG-SFP (× 35 magnification) and (**d**) LG-SFP (× 70 magnification).

The microstructures of SFP and LG-SFP were examined with a scanning electron microscope (SEM). The obtained images in Figure 1 show that, before the vacuum-based impregnation, the morphology of SFP particles is inhomogeneous; they are rough with visible smaller particles or dust located on their surface. After the impregnation of SFP with LG, the particles seem inhomogeneous as well but with slightly smoother and uniform surfaces and sharp edges.

**Figure 2:**
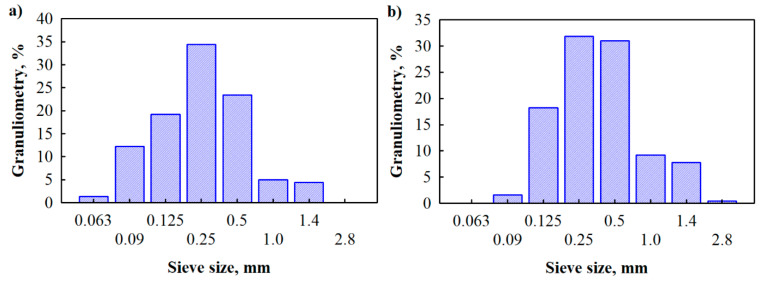
Granuliometric composition of: (**a**) SFP particles and (**b**) LG-SFP particles.

Energy dispersion spectroscopy (EDS) results in Figure 1d show that in the case of LG impregnation, LG-SFP particles have 4.3% sodium and 4.5% silica which is the result of vacuum impregnation of LG. It is also determined (Figure 2) that the size of non-impregnated SFP ranges from 0.063 mm up to 1.4 mm with a residual moisture content of 1.1 ± 0.2 wt.% and bulk density of 527 ± 2 kg/m^3^, while LG-SFP particles have particle sizes ranging from 0.09 mm up to 2.8 mm with a residual moisture content of 0.50 ± 0.1 wt.% and a bulk density of 531 ± 2 kg/m^3^.

It is known that at higher temperatures, such as 150 °C and more, LG swells, thus increasing its volume from 50 to 70 times, forming a highly porous structure [22]. As can be seen from Figure 1c,d, when exposed to LG and hardening temperature, some LG-SFP particles appear as expanded bubbles that form conglomerates thus increasing the amount of larger particles (Figure 2).

**Figure 3:**
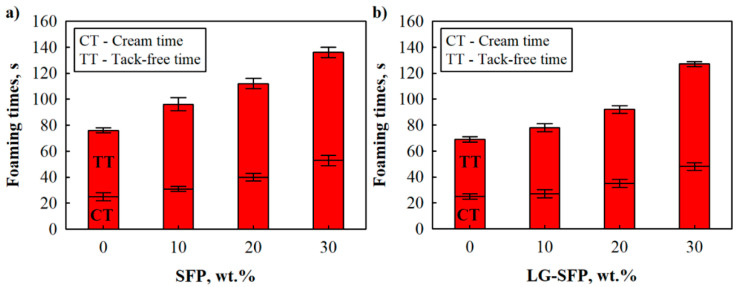
Foaming times of unmodified, SFP, and LG-SFP modified PUR foam mixtures: (**a**) SFP and (**b**) LG-SFP.

The foaming behaviour of SFP and LG-SFP modified PUR mixtures was evaluated by determining cream and tack-free times. The cream time was measured from the beginning of raw materials mixing to a visible start of the reaction, bubble formation, and the change in mixture colour, while tack-free time was determined as the time when the foam surface was no longer tacky according to [13], Annex E. Based on the results in Figure 3, SFP and LG-SFP particles slow down the foaming process of modified PUR foams. Compared to PUR-REF (unmodified), SFP particles extend cream time by 6 s, 15 s, and 28 s, while tack-free time increases by 20 s, 36 s, and 66 s at 10 wt.%, 20 wt.%, and 30 wt.% SFP loadings, respectively.

Moreover, LG-SFP increased cream time by 2 s, 10 s, and 23 s, while tack-free time was extended by 9 s, 23 s, and 58 s at 10 wt.%, 20 wt.%, and 30 wt.% LG-SFP loadings, respectively. As expected, the addition of SFP and LG-SFP particles into the PUR mixture negatively affected the individual foaming times. According to [23], this might be due to increased acidity of the mixture, which slows down the reaction rate of the raw components.

However, compared to SFP particles, LG-SFP particles slightly improved the cream and tack-free times. This is unexpected because most of the studies regarding PUR composite foams indicate slower reactivity of filler-modified systems.

This improvement indicated that the SFP impregnation with LG avoided the agglomeration of the particles; they had a better distribution in the final PUR foams, while the addition of unmodified SFP particles resulted in the formation of conglomerates and increased the dynamic viscosity of PUR mixtures (Table 2). Similar observations and conclusions were made in several other studies [24,25].

Additionally, as the authors of [26] observed, the improvements of one filled mixture compared to another filled mixture may be attributed to the different chemical interactions between certain particulate fillers and isocyanate, which are related to different chemical compositions and particle size distributions of the used fillers.

Previous studies showed that some PUR foam compositions are characterised by dimensional instabilities and structural damage when polyols with lower functionalities are incorporated [27,28], or lower indices of isocyanate are used [29]. It was also observed that the addition of fillers into low functionality PUR systems might also affect the dimensional stability at elevated and reduced temperatures [30].

The results of dimensional stability at 70 °C/90% and −20 °C temperatures (Table 3) show that the linear changes are similar for all PUR foam compositions regardless of the type of filler, its modification, or amount. Compared to PUR-REF, the addition of unmodified SFP-10 resulted in slightly lower dimensional changes at 70 °C, while dimensional stability at −20 °C was better for LG-SFP filled PUR foams at 10 wt.% and 20 wt.% loadings. According to harmonised standards for in situ formed sprayed rigid polyurethane and polyisocyanurate foam products [13], the maximum allowable dimensional changes at 70 °C/90% should not exceed 15% for length and width, and 10% for thickness, while at −20 °C they should not be higher than 3% for length, width, and thickness. The studied results in Table 3 show that SFP and LG-SFP modified PUR foams are characterised by sufficient dimensional performance. Similar observations were reported in casein/apricot filler modified PUR foam composites [31]. However, the authors of [32] determined that the incorporation of keratin fibres increased the dimensional stability of PUR foams while Fyrol slightly deteriorated the mentioned parameter. Such differences might be associated with the cellular structure, i.e., average cell size, the thickness of cell walls, closed-cell content and, of course, the blowing agent used.

During immersion in water, the short-term water absorption, expressed as kilograms per square metre of all foams, was evaluated by the weight gain after immersion in water for 24 h. This characteristic is essential because it is important that PUR foam does not show a propensity to absorb water because it changes the thermal insulation properties of the product which is to be installed in buildings [33].

**Figure 4:**
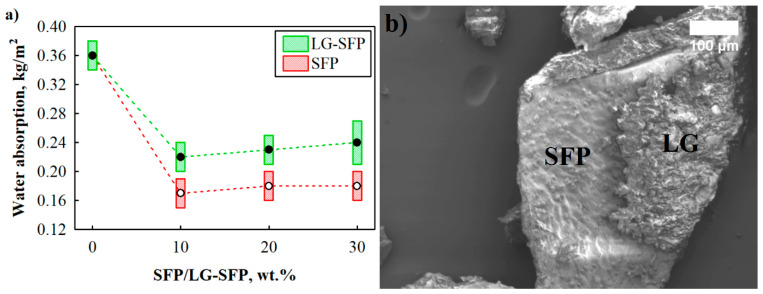
Water absorption of PUR-REF, SFP and LG-SFP modified PUR foams: (**a**) short-term water absorption and (**b**) LG-SFP particle and bonding boundary of oil and LG.

As can be seen from Figure 4a, SFP particles greatly reduce water absorption of modified PUR foams. All SFP modified PUR foam compositions had 50% or greater reduction in the mentioned parameter. According to [34], sunflower press cake has a residual oil content of up to 18%, which due to it being a semi-drying oil, slowly hardens during thermal treatment at 110 °C, thus forming a softer finish on the particle surface. This small fraction of oil repels water and prevents water molecules from penetrating the cellular structure of particles and increasing the water absorption of SFP modified PUR foams. It was reported that the cellular structure affected the water absorption ability of the PUR foams, i.e., more water can be accommodated by larger cells [35]. This observation is in good agreement with the results presented in Table 4. PUR-REF foam is characterised by larger average cell size and lower content of closed cells which impact the overall increase in water absorption compared to SFP modified PUR foams.

Interesting observations were made for LG-SFP modified PUR foams. LG-SFP-10 reduced water absorption by 39%, while LG-SFP-20 and LG-SFP-30 reduced it by 36% and 33%, respectively. For instance, comparing the results of SFP-30 and LG-SFP-30, it can be noticed that LG-SFP-30 has greater closed-cell content; however, water absorption results show an increment. Apparently, the modification of SFP with LG was not successful due to the fact that LG does not properly harden when mixed with oil. As a result, insufficient bonding between oil and LG layers on SFP particles is formed, as can be seen from Figure 4b.

The addition of SFP and LG-SFP particles resulted in an apparent density increase (Table 4). SFP-10, SFP-20 and SFP-30 modified PUR foams had a 5%, 69% and 120% increase in the mentioned parameter, respectively, while LG-SFP-10, LG-SFP-20 and LG-SFP-30 had increases of 8%, 38% and 74%, respectively.

The data in previous studies of filler reinforced polymeric foams show that the incorporation of particles, such as expandable graphite [36] or micro-cellulose [37], leads to higher apparent density values, which are directly connected to lower reactivity of the PUR foam systems and higher density of the filler particles. Interestingly, the values of apparent density of PUR foams with LG modified SFP particles are somewhat lower, and the microstructure is more uniform with the higher number of closed cells compared to PUR foams with unmodified SFP particles.

Even though the bulk density of LG-SFP particles is 4 kg/m^3^ higher compared to SFP particles, the difference is quite low and the increased apparent density value for SFP particles modified foams can be attributed to the rheological properties of PUR premixes. For instance, PUR foam with SFP-20 has almost the same apparent density as LG-SFP-30 modified foam. The authors of [38] studied the impact of plum stone filler, silanized plum stone filler, and [3] algal cellulose on the performance of PUR foams and concluded that it could be attributed to the greater initial dynamic viscosity of PUR foams with unmodified filler particles.

**Figure 5:**
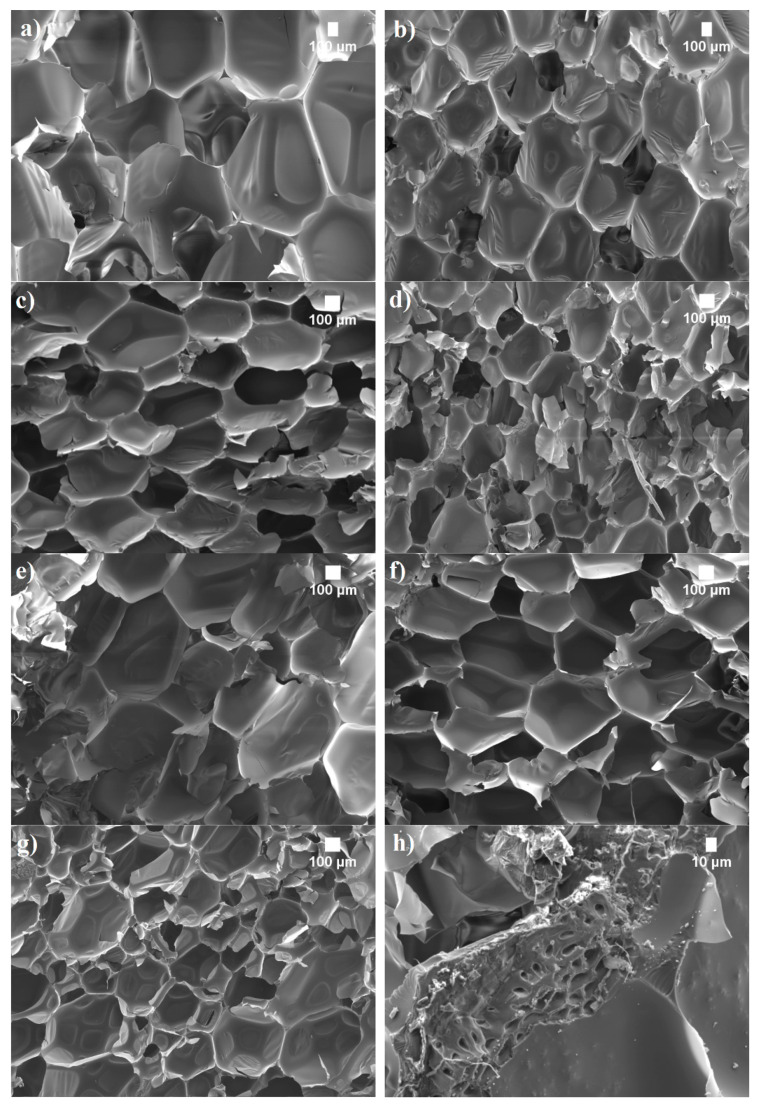
Microstructure of SFP and LG-SFP modified PUR foams (×50 magnification): (**a**) PUR-REF; (**b**) SFP-10; (**c**) SFP-20; (**d**) SFP-30; (**e**) LG-SFP-10; (**f**) LG-SFP-20; (**g**) LG-SFP-30 and (**h**) cut section of SFP particle in cell struts (×400 magnification).

The impact of SFP and LG-SFP on the cellular structure of PUR foams was evaluated by SEM. As shown in Figure 5, the obtained PUR foams have a typical microstructure with a high content of closed cells (Table 4). In addition, when unmodified SFP particles are incorporated into PUR foam structure, the average cell size reduces from 566 μm for PUR-REF to 416 μ, 364 μm and 272 μm for SFP-10, SFP-20, and SFP-30, respectively, and to 421 μm, 273 μm and 227 μm for LG-SFP-10, LG-SFP-20 and LG-SFP-30 PUR foams, respectively. The decrease in cell size in SFP and LG-SFP modified PUR foam is a result of higher dynamic viscosity compared to PUR-REF, and the filler’s ability to limit the growth of cells and act as a nucleation centre during the foaming process [39,40]. Compared to PUR-REF, the application of SFP and LG-SFP resulted in the formation of a higher content of closed cells. It can be assumed that sufficient compatibility between filler particles and the PUR foam matrix was achieved, although a study [37] reported that the addition of filler particles in PUR foams resulted in the formation of irregular structures with lower content of closed cells, that might be due to reduced reactivity which affected the cell opening tendency.

As for the thermal conductivity of modified PUR foams (Table 4), it is significantly impacted by the chemical and physical properties of the raw materials, the average cell size, and the closed-cell content of the resultant PUR foam [41,42]. In the current study, the effect induced by the SFP and LG-SFP is an overall reduction of the thermal conductivity of modified PUR foams. For example, compared to PUR-REF, SFP-10, SFP-20, and SFP-30 had a decrease in thermal conductivity by 9%, 17%, and 9%, respectively, while LG-SFP-10, LG-SFP-20, and LG-SFP-30 showed decreases of 5%, 7%, and 10%, respectively. Greater improvements of thermal conductivity values are observed for SFP modified PUR foams due to the fact that LG-SFP particles probably have greater thermal conductivity as they were modified with LG.

**Figure 6:**
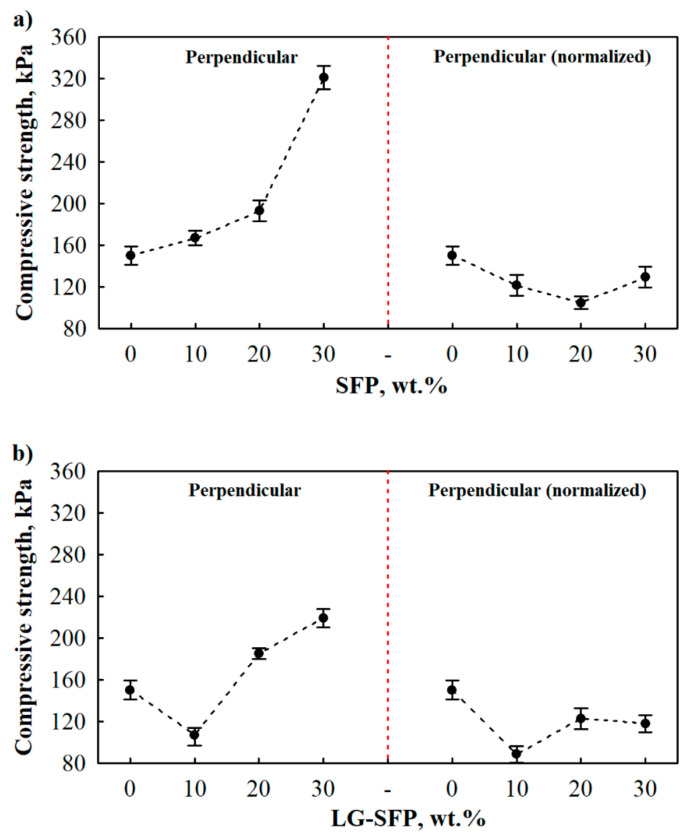
Compressive strength of SFP and LG-SFP modified PUR foams: (**a**) SFP modified PUR foam perpendicularly to foaming direction; (**b**) LG-SFP modified PUR foam perpendicularly to foaming direction.

**Figure 7:**
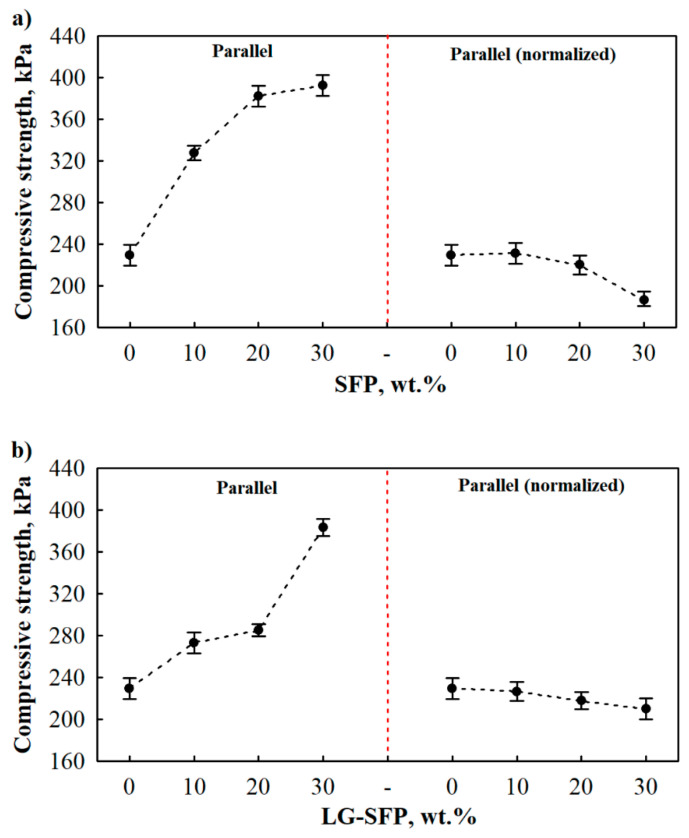
Compressive strength of SFP and LG-SFP modified PUR foams: (**a**) SFP modified PUR foam parallel to foaming direction and (**b**) LG-SFP modified PUR foam parallel to foaming direction.

The compressive strength of unmodified PUR-REF, SFP, and LG-SFP modified PUR foams are reported in Figure 6 and Figure 7. The effect of SFP and LG-SFP addition on mechanical behaviour was evaluated in terms of compressive strength both perpendicular and parallel to the foaming direction. Additionally, normalised compressive strength to 35 kg/m^3^ apparent density was calculated in both directions.

As can be seen from Figure 6a (left part of the Figure), SFP particles positively affected the compressive strength of modified PUR foams perpendicular to the foaming direction. SFP increased the parameter by 11%, 28%, and 114%, respectively, at 10 wt.%, 20 wt.%, and 30 wt.% SFP loadings. Moreover, a similar tendency was observed for LG-SFP modified PUR foams (Figure 6b (left part of the Figure)). The greatest increase was observed for LG-SFP-30 which was by 46% compared to PUR-REF foam.

However, normalised perpendicular to the foaming direction compressive strength decreased with the addition of SFP and LG-SFP particles (Figure 6a,b (right part of the Figure). When SFP and LG-SFP modified PUR foam was produced at an apparent density of 35 kg/m^3^, their perpendicular to the foaming direction compressive strength was reduced by 19%, 30%, and 14% at 10 wt.%, 20 wt.%, and 30 wt.% SFP loading, respectively, and by 41%, 18%, and 21% at 10 wt.%, 20 wt.%, and 30 wt.% LG-SFP loadings, respectively.

Similar observations were conducted in parallel to the foaming direction compressive strength (Figure 7). These showed that SFP particles increased the mechanical parameter by 43%, 67%, and 71% at 10 wt.%, 20 wt.%, and 30 wt.% SFP loadings, respectively, (Figure 7a (left part of Figure)) while LG-SFP increased them by 19%, 24%, and 67% at 10 wt.%, 20 wt.%, and 30 wt.% LG-SFP loadings, respectively (Figure 7b (left part of the Figure)). The improvement in compressive strength in both directions may be explained by the cellular structure of modified PUR foams. As shown in Table 4, PUR foams modified with a higher amount of SFP and LG-SFP particles possessed a closed-cell structure compared to PUR-REF foam. This provided support to withstand the compressive load. The reinforcing effect can also be included because it provided a stronger interface between SFP, LG-SFP particles, and the polymer matrix (Figure 5h).

The tendency of reduced normalised compressive strength can also be observed from Figure 7a,b (right part of the Figure). SFP and LG-SFP particles negatively impacted the compressive strength normalised parallel to the foaming direction and decreased it for all amounts of particles. Of course, the highest drop in compressive strength value was observed for SFP-30 and LG-SFP-30 PUR foams, i.e., 19% and 9%, respectively.

Irrespective of the foaming direction, to obtain 35 kg/m^3^ apparent density of SFP and LG-SFP modified PUR foams, a higher amount of blowing agent should be used. This way, the average cell size would increase, and cell walls would become thinner and weaker to withstand the compressive loading. As reported in previous studies by [43], cell morphology is an important factor that impacts the overall mechanical performance of PUR and modified PUR foams.

Regardless of the filler type, the compressive strength values in the direction perpendicular to the foam rise direction are lower than the values for the parallel direction. The difference is explained by the cell anisotropy. The elongated cells in the compression directions cause greater values of the compressive strength than in the parallel direction, but the parameter in the perpendicular direction is lower, as was observed earlier by [25].

**Figure 8:**
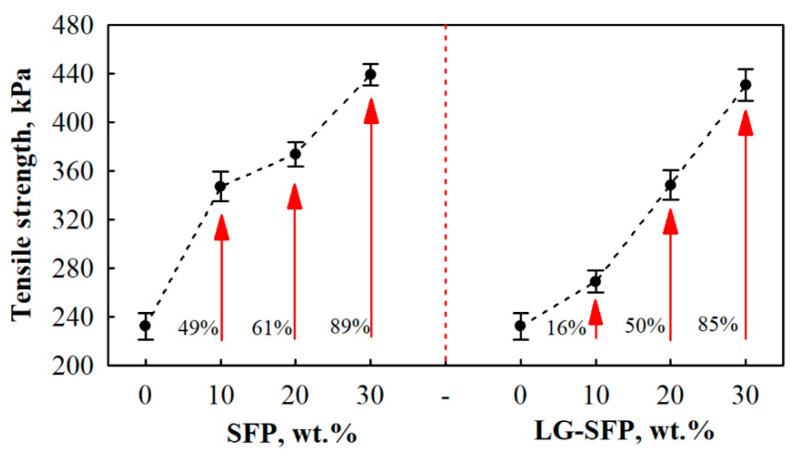
Tensile strength of SFP and LG-SFP modified PUR foams.

Figure 8 presents the results of tensile strength. The addition of SFP and LG-SFP particles positively affects PUR foams. SFP particles increase the tensile strength by 49%, 61%, and 89% at 10 wt.%, 20 wt.%, and 30 wt.% SFP loadings, respectively, while LG-SFP increase it by 16%, 50%, and 85% at 10 wt.%, 20 wt.%, and 30 wt.% LG-SFP loadings, respectively. The improvement suggests a sufficient strength of the interfacial interactions between the SFP, LG-SFP, and PUR matrix. It was particularly pronounced when SFP was used because of the shape and oil fraction on the surface of the particles, which, together with the reduced average cell size, promotes the tensile strength by the facilitated stress distribution [44]. Even though the tensile strength of LG-SFP particles modified PUR foams increases, the rise is lower compared to SFP particles modified PUR foams. This can be attributed to the fact that vegetable oil does not allow for LG to fully harden, thus forming insufficient bonding between oil and LG layers (Figure 4b).

**Figure 9:**
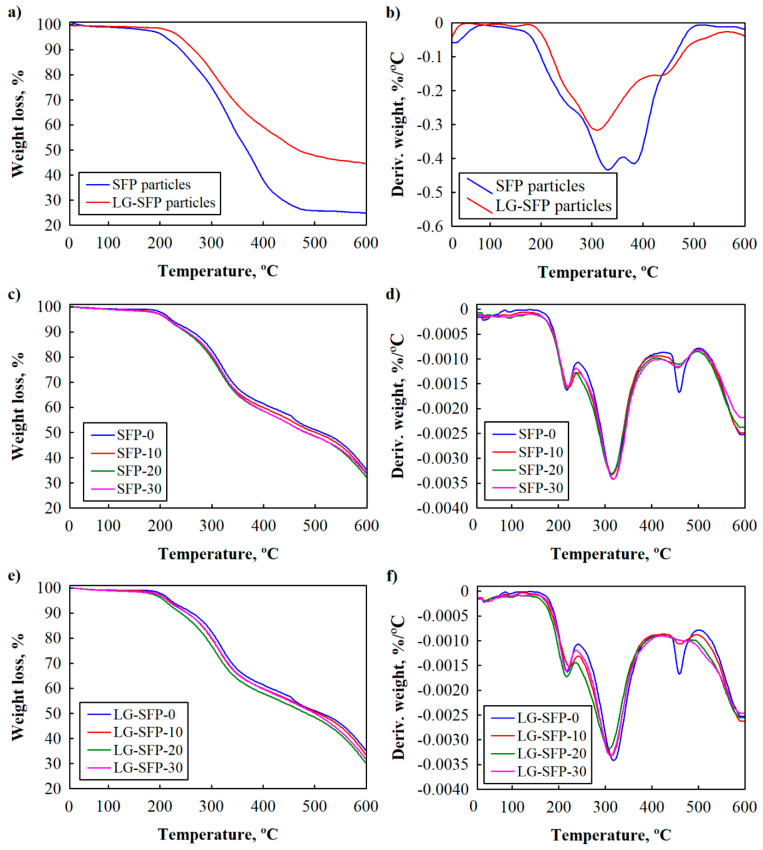
Thermal stability of SFP particles, LG-SFP particles, PUR-REF, SFP and LG-SFP modified PUR foams: (**a**), (**c**) and (**e**) TGA and (**b**), (**d**) and (**f**) DTG.

In order to determine the impact of SFP and LG-SFP particles and their amounts on the thermal stability of resulting PUR foams, thermogravimetric (TGA) and derivative thermogravimetric (DTG) measurements were performed (Figure 9).

Two stages of thermal degradation of SFP and LG-SFP particles may be observed (Figure 9a,b). The first and the second peaks are at 330 °C and 390 °C, respectively, are overlapped and are associated to the degradation of protein and lignin in SFP particles [45]. However, a slower degradation rate of complex substances is noticed for LG-SFP particles due to the presence of the protective layer of LG. It is determined that LG degrades at a temperature interval of (150–300) °C [46]; therefore, the main peak of LG overlaps the two main peaks of SFP. It can also be observed from Figure 9a that LG-SFP particles are characterised by a higher char yield at 600 °C, i. e. 44%, which is almost twice as high than for SFP particles (char yield 24%).

Three stages of thermal degradation of modified PUR foams may be observed (Figure 9c–f). The first takes place at a temperature interval of (150–250) °C with a weight loss of 12%. It is related to the dissociation of the urethane bonds, which correspond to the degradation of the rigid segments [47,48] and degradation of small fraction of LG in LG-SFP particles. The second stage is followed by 55% weight loss and begins between 300 °C and 350 °C. It corresponds to the thermal decomposition of the soft segments of two polyols used in PUR-REF, SFP, and LG-SFP modified PUR foams [49], protein and lignin in SFP and LG-SFP particles. Lastly, the third stage of degradation with the weight loss of approximately 70% is in the temperature range of (500–600) °C. This stage is associated with the decomposition of the fragments formed in the previous degradation stages [50].

Table 5 presents the results of temperature values of corresponding decomposition stages at weight losses of 5% and 50%. Compared to PUR-REF foam, SFP particles modified foams require lower temperatures to go between the degradation stages. However, LG-SFP particles modified PUR foams exhibited a slightly higher temperature at the third stage compared to PUR-REF foam. It is noticeable that T_5%_ for LG-SFP particles modified foams does not differ much compared to SFP particles modified foams, but a slight increase in temperatures T_5%_ and T_50%_ can be observed. It means that LG-SFP particles are capable of shifting degradation to higher temperatures compared to SFP particles because LG acts as an additional barrier. This behaviour was also observed in other studies [51,52]

Additionally, Figure 9f shows that the degradation rate of LG-SFP particles modified PUR foams is slower compared to PUR-REF and SFP particles modified foams. For instance, the degradation rate of PUR-REF and SFP-30 is 0.0034 %/°C, while for LG-SFP-30 it is 0.0032 %/°C, and for LG-SFP-20 it is 0.0030 %/°C. This can be attributed to the assumption that filler particles in composite systems absorb part of the heat generated during the decomposition process [53].

Analysing the impact of SFP and LG-SFP particles modified PUR foams on the thermal stability, the char yield at 600 °C was also determined. However, SFP and LG-SFP particles modified PUR foams do not show a higher char yield than PUR-REF. The more SFP or LG-SFP particles are added into the PUR foam formulation, the lower the observed char yield is.

## 4. Conclusions

The current paper presents the impact of SFP and LG-SFP particles as fillers on the physical and mechanical properties of PUR foams. The study showed that the incorporation of unmodified and modified sunflower press cake particles into PUR foam formulations affected the rheological properties of polyol premixes. It was also determined that rheological properties affected the resultant microstructure of modified PUR foams in which the incorporation of SFP and LG-SFP particles led to the formation of foams with higher density, irregular cell network, and an increase in closed-cell content. Such changes influenced the performance characteristics of modified PUR foams, such as thermal conductivity, dimensional stability, short-term water absorption, compressive and tensile strengths, and thermal stability. The highest impact on thermal conductivity was observed for SFP-20 and LG-SFP-30, which reduced the parameter by 17% and 10%, respectively, due to increased closed-cell content and more regular structure. SFP and LG-SFP particles allowed modified PUR foams with sufficient structural and dimensional stability at extreme conditions to be obtained. With the addition of SFP and LG-SFP particles, compressive strength in both directions significantly improved, i.e., maximum increases of 114% and 46%, respectively, were observed for the perpendicular direction, and 71% and 67%, respectively, for the parallel direction. On the other hand, after the normalisation of compressive strength to 35 kg/m^3^ apparent density, no improvements were observed. Compared to PUR-REF foam, the addition of SFP and LG-SFP particles increased tensile strength by 89% and 85%, respectively. Moreover, short-term water absorption by partial immersion was reduced by almost half, which showed that SFP and LG-SFP particles had greater hydrophobicity compared to PUR-REF foams. Additionally, thermal stability measurements showed that LG-SFP particles partially act as a barrier and shift temperatures at 5% and 50% weight loss to higher values.

## Figures and Tables

**Table 1:** Compositions of PUR-REF, SFP, and LG-SFP particles modified PUR foams.

Materials	Content, pbw		
	PUR-REF Foam	SFP Modified PUR Foam	LG-SFP Modified PUR Foam
BioPolyol RD	40	40	40
Petol PZ 400-4G	60	60	60
Distilled water	2.7	2.7	2.7
Polycat 9	1	1	1
ST-52	3	3	3
Particles, wt.%	0	10; 20; 30 ^(1)^	10; 20; 30 ^(1)^
Isocyanate indice	1.25	1.25	1.25

^(1)^ The number of particles is presented in wt.%. The number of SFP and LG-SFP particles in the whole mixture volume is 12 vol.%, 24 vol.% and 36 vol.%. The number of SFP and LG-SFP particles in PUR foam product is 0.62 vol.%, 1.5 vol.% and 4.0 vol.%.

**Table 2:** Dynamic viscosity of PUR-REF, SFP, and LG-SFP modified PUR mixtures.

Sample	Dynamic Viscosity, mPa·s
PUR-REF	115 ± 5
SFP-10	130 ± 3
SFP-20	275 ± 4
SFP-30	400 ± 6
LG-SFP-10	125 ± 5
LG-SFP-20	265 ± 4
LG-SFP-30	387 ± 6

**Table 3:** Dimensional stability of PUR-REF, SFP, and LG-SFP modified PUR foams.

Sample	Dimensional Stability at 70 °C and 90%, %			Dimensional Stability at −20 °C, %		
	Length	Width	Thickness	Length	Width	Thickness
PUR-REF	1.2 ± 0.1	1.3 ± 0.1	1.5 ± 0.1	0.9 ± 0.1	0.5 ± 0.1	0.8 ± 0.1
SFP-10	1.1 ± 0.1	1.3 ± 0.1	1.7 ± 0.1	0.8 ± 0.1	0.7 ± 0.1	0.9 ± 0.1
SFP-20	1.2 ± 0.1	1.5 ± 0.1	1.8 ± 0.1	0.8 ± 0.1	0.7 ± 0.1	1.0 ± 0.1
SFP-30	1.4 ± 0.1	1.7 ± 0.1	1.9 ± 0.1	0.9 ± 0.1	0.8 ± 0.1	1.0 ± 0.1
LG-SFP-10	1.3 ± 0.1	1.2 ± 0.1	1.3 ± 0.1	0.7 ± 0.1	0.5 ± 0.1	0.8 ± 0.1
LG-SFP-20	1.4 ± 0.1	1.5 ± 0.1	1.5 ± 0.1	0.8 ± 0.1	0.5 ± 0.1	0.8 ± 0.1
LG-SFP-30	1.4 ± 0.1	1.6 ± 0.1	1.6 ± 0.1	0.9 ± 0.1	0.7 ± 0.1	1.0 ± 0.1

**Table 4:** Apparent density and microstructural characteristics of SFP and LG-SFM modified PUR foams.

Sample	Characteristic			
	Apparent Density, kg/m^3^	Thermal Conductivity, W/(m·K)	Closed-Cell Content, vol.%	Cell Size, μm
PUR-REF	39 ± 3	0.0354 ± 0.0003	81 ± 2	566 ± 12
SFP-10	41 ± 4	0.0322 ± 0.0004	87 ± 2	416 ± 24
SFP-20	66 ± 5	0.0294 ± 0.0002	90 ± 1	364 ± 15
SFP-30	86 ± 5	0.0321 ± 0.0003	85 ± 4	272 ± 22
LG-SFP-10	42 ± 3	0.0336 ± 0.0003	88 ± 3	421 ± 13
LG-SFP-20	54 ± 4	0.0328 ± 0.0004	90 ± 2	273 ± 16
LG-SFP-30	68 ± 6	0.0319 ± 0.0002	92 ± 2	227 ± 10

**Table 5:** Thermal degradation parameters of PUR-REF, SFP, and LG-SFP modified PUR foams.

Sample	T_5%_, °C	T_50%_, °C	T_max_, °C			Char Yield, %
			1st Stage	2nd Stage	3rd Stage	
PUR-REF	221	513	219	319	459	35.3
SFP-10	217	499	223	317	457	34.0
SFP-20	213	481	219	319	461	32.3
SFP-30	215	479	219	317	457	33.7
LG-SFP-10	217	505	225	313	463	33.6
LG-SFP-20	216	483	217	309	467	30.3
LG-SFP-30	215	499	217	313	467	31.9

## Data Availability

Data sharing not applicable.
